# Supplementary material for: mHealth Interventions to Address Physical Activity and Sedentary Behavior in Cancer Survivors: A Systematic Review
Source: Int J Environ Res Public Health. 2021 May 28;18(11):5798. doi: 10.3390/ijerph18115798 (PMC8198944; doi:10.3390/ijerph18115798)
Supplement: Supplementary file 1 [file ijerph-18-05798-s001.zip › Supplementary S1.pdf]

Search strategy

Limit: 2000 - 13 April 2020

‘cancer’ population
cancer OR neoplasm OR tumour OR carcinoma OR malignancy
‘mHealth’ intervention method
app* OR ‘mobile health’ OR mHealth OR ‘electronic health’ OR eHealth OR smartphone OR ‘mobile phone’ OR ‘cell phone’ OR internet OR wearable* OR tracker* OR technology OR teleHealth OR telerehabilitation OR telemedicine OR ‘short message service’ OR SMS OR ‘multimedia messaging service’ OR MMS OR ‘text message’
‘physical activity’ or ‘sedentary behaviour’ outcome variable
exercise OR ‘physical exercise’ OR ‘physical activit*’ OR ‘exercise therap*’ OR ‘motor function’ OR ‘motor activit*’ OR kinesiotherapy OR ‘aerobic exercise’ OR run* OR jog* OR walk* OR sport* OR fitness OR workout OR ‘outdoor activit*’ OR ‘active transport’ OR ‘movement therapy’ OR ‘physical fitness’ OR sedentar* OR ‘sedentary behaviour’ OR sit* OR inactive* OR recline* OR stationary OR seated OR screen*
