# Supplementary material for: mHealth Interventions to Address Physical Activity and Sedentary Behavior in Cancer Survivors: A Systematic Review
Source: Int J Environ Res Public Health. 2021 May 28;18(11):5798. doi: 10.3390/ijerph18115798 (PMC8198944; doi:10.3390/ijerph18115798)
Supplement: Supplementary file 1 [file ijerph-18-05798-s001.zip › Supplementary S2.pdf]

Risk of Bias Summary

RCT studies*

First Author	Randomization process	Deviations from intended interventions	Missing outcome data	Measurement of the outcome	Selection of the reported result	Overall judgement
Cadmus-Bertram [42]	+	+	+	+	+	+
Gell [44]	+	+	+	+	?	?
Haggerty [48]	+	+	?	-	+	-
Hartman [52]	+	+	+	+	+	+
Kenfield [47]	+	+	+	+	+	+
Lynch [38,39]	+	+	+	+	+	+
Maxwell-Smith [56]	+	+	+	+	+	+
Mayer [46]	?	+	+	+	+	?
McNeil [61]	+	+	+	+	+	+
Mendoza [40]	+	+	+	+	+	+
Ormel [65]	+	+	+	?	+	?
Pope [49]	+	+	+	+	+	+
Singh [53]	+	+	+	?	+	?
Van Blarigan [69]	+	+	+	+	+	+
Valle [51]	+	+	+	+	+	+
Villaron [64]	?	+	+	+	?	-

Non RCT**

First Author	Bias due to confounding	Bias in selection of participants	Bias in classification of interventions	Bias due to deviations from intended interventions	Bias due to missing data	Bias in measurement of outcomes	Bias in selection of the reported result	Overall judgement
Cheong [59]	?	/	/	/	?	-	+	-
Chung [58]	N/I	/	+	+	/	-	+	N/I
Delrieu [63]	-	/	+	+	/	-	+	-
Gell [43]	N/I	/	+	+	+	-	+	N/I
Götte [66]	?	/	/	+	+	-	+	-
Kim [60]	N/I	/	+	+	?	-	+	N/I
Le [41]	N/I	/	/	/	/	-	+	N/I
Lozano-Lozano [67]	N/I	/	+	+	+	-	+	N/I
McCarroll [50]	N/I	/	+	+	+	-	+	N/I
Ovans [45]	?	/	+	+	?	-	+	-
Puszkiewic [68]	?	/	+	+	+	-	+	-
Short [55]	N/I	/	+	N/I	?	-	+	N/I
Spark [54]	?	/	+	+	/	-	+	-
Trinh [62]	N/I	/	+	+	/	-	+	N/I
Uhm [57]	/	/	+	+	+	?	+	?

*Assessed using RoB2.0; low risk of bias (+); some concerns (?); high risk of bias (-)
**Assessed using ROBINS-I; low risk of bias (+), moderate risk of bias (/), serious risk of bias (?), critical risk of bias (-), No information (N/I)
